# Targeting dermatophyte Cdc42 and Rac GTPase signaling to hinder hyphal elongation and virulence

**DOI:** 10.1016/j.isci.2024.110139

**Published:** 2024-05-28

**Authors:** Masaki Ishii, Yasuhiko Matsumoto, Tsuyoshi Yamada, Hideko Uga, Toshiaki Katada, Shinya Ohata

**Affiliations:** 1 Research Institute of Pharmaceutical Sciences, Faculty of Pharmacy, Musashino University, Tokyo 202-8585, Japan; 2 Department of Microbiology, Meiji Pharmaceutical University, 2–522–1 Noshio, Kiyose, Tokyo 204–8588, Japan; 3 Teikyo University Institute of Medical Mycology, Teikyo University, Hachioji, Tokyo 192-0395, Japan; 4 Asia International Institute of Infectious Disease Control, Teikyo University, Tokyo 173-0003, Japan

**Keywords:** molecular biology, Microbiology, Mycology, cell biology

## Abstract

The development of antifungal drugs requires novel molecular targets due to limited treatment options and drug resistance. Through chemical screening and establishment of a novel genetic technique to repress gene expression in *Trichophyton rubrum*, the primary causal fungus of dermatophytosis, we demonstrated that fungal Cdc42 and Rac GTPases are promising antifungal drug targets. Chemical inhibitors of these GTPases impair hyphal formation, which is crucial for growth and virulence in *T. rubrum*. Conditional repression of Cdc24, a guanine nucleotide exchange factor for Cdc42 and Rac, led to hyphal growth defects, abnormal cell morphology, and cell death. EHop-016 inhibited the promotion of the guanine nucleotide exchange reaction in Cdc42 and Rac by Cdc24 as well as germination and growth on the nail fragments of *T. rubrum* and improved animal survival in an invertebrate infection model of *T. rubrum*. Our results provide a novel antifungal therapeutic target and a potential lead compound.

## Highlights

• A screen of small GTPase inhibitors identifies Cdc42/Rac as a novel antifungal target
• We identified compounds that inhibit *T. rubrum* Cdc24-mediated Cdc42/Rac activation
• Genetic repression of Cdc42/Rac pathway in *T. rubrum* markedly impairs hyphal formation
• Fungal Cdc42/Rac inhibitor alleviates infection in an animal model and growth on nails

## Graphical abstract

**Figure:**
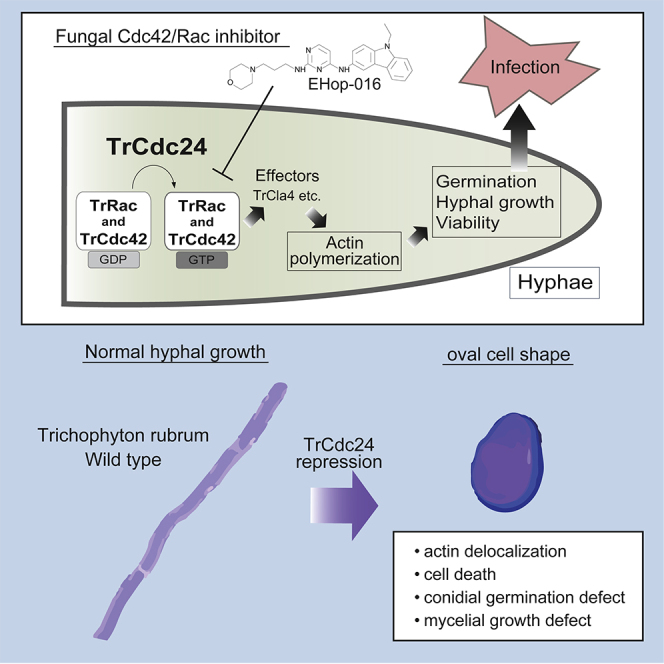

## Introduction

Dermatophytes are infectious fungi that cause dermatophytosis, a condition that affects over 100 million people worldwide.^1^^,^^2^^,^^3^^,^^4^ Despite the existence of antifungal treatments, dermatophytosis often proves to be stubborn and recurrent, particularly in the case of tinea unguium, a nail infection caused by dermatophytes.^5^ Dermatophytes have the ability to invade superficial host tissues, such as hair and skin, and in rare instances, can infect dermal tissues and lymph nodes in immunocompromised patients.^5^ Conditions such as dermatophytosis and onychomycosis can lead to severe asthma and foot ulcers in patients with diabetes mellitus.^6^^,^^7^ The current antidermatophytic drugs target a limited number of fungal enzymes involved in the synthesis of ergosterol, such as squalene epoxidase (allylamine and benzylamine classes) and sterol 14α-demethylase (azole class).^8^ However, the emergence of drug-resistant strains of dermatophytes underscores the urgent need for the discovery of new therapeutic targets.^9^^,^^10^^,^^11^

To establish an infection, fungal spores adhere to the superficial layer of the host’s skin, germinate, form hyphae through cell proliferation and morphological elongation, and penetrate the superficial tissue. Pathological observations, reconstructed skin experiments, and *ex vivo* analysis suggest that conidial germination and hyphal growth are critical steps in fungal colonization and infection.^12^^,^^13^ In *Trichophyton rubrum*, the most common causative agent of dermatophytosis,^1^ genes related to drug resistance and pH response have been identified.^9^^,^^14^^,^^15^^,^^16^^,^^17^ When knocked out, genes essential for proliferation and hyphal growth result in fungal death, thereby preventing analysis. Therefore, conditional expression strains that express genes only under certain environmental conditions are used. However, such technology is not yet established in *T. rubrum*, and genes involved in hyphal growth remain largely unidentified.

Small GTPases function as molecular switches in intracellular signaling, altering their conformation based on the guanine nucleotide forms they bind.^18^ Small GTPases play a key role in conidial germination and hyphal growth in human pathogenic fungi.^19^^,^^20^ For instance, Rac1 and Rac2 are synthetically essential for hyphal growth in *Cryptococcus neoformans*, and Arl1 is a crucial protein for invasive filamentous growth in *Candida albicans*. The Rho family, a well-studied and conserved small GTPase family, activates its effectors to regulate cell morphology and growth by modulating actin organization in mammals.^21^ In *Saccharomyces cerevisiae*, the Rho family small GTPase Cdc42 is vital for growth and viability,^22^^,^^23^^,^^24^ whereas co-deletion of both Cdc42 and another Rho family protein Rac results in cell death in the filamentous fungi *Ustilago maydis*, *Aspergillus nidulans*, and *Aspergillus niger*.^25^^,^^26^^,^^27^ In *Neurospora crassa* and *Claviceps purpurea*, the DH-PH domain-containing protein Cdc24 activates Cdc42 and Rac by promoting the exchange of GDP for GTP bound to Cdc42 and Rac as a guanine nucleotide exchange factor (GEF).^28^^,^^29^ Since Cdc24 is an essential protein and a modulator of hyphal formation in some fungi,^28^^,^^29^^,^^30^^,^^31^^,^^32^ the activation machinery of Cdc42 and Rac by Cdc24 could be a novel therapeutic target for the development of antifungal agents. However, the function of these proteins in dermatophytes is unknown, and chemical compounds that inhibit GEF-mediated fungal Cdc42 and Rac activation have not been identified.

In the present study, we established a method to suppress gene expression in *T. rubrum* in a copper-dependent manner. We discovered that both chemical and genetic inhibition of the Cdc24-Cdc42/Rac signaling pathway significantly impeded conidial germination and hyphal formation in *T. rubrum*. EHop-016, an inhibitor of the interaction between Cdc24 and Cdc42/Rac, inhibited fungal growth on nail fragments and reduced the lethality of silkworms injected with *T. rubrum*. Our results propose that targeting small GTPase signaling, especially the Cdc24-Cdc42/Rac pathway, holds promise as a therapeutic approach for developing antifungal agents. In addition, EHop-016 can serve as a lead compound for the development of antifungal drugs.

## Results

### Mammalian Rac and Rac/Cdc42 inhibitors suppress the conidial germination of *T. rubrum*

**Figure 1:**
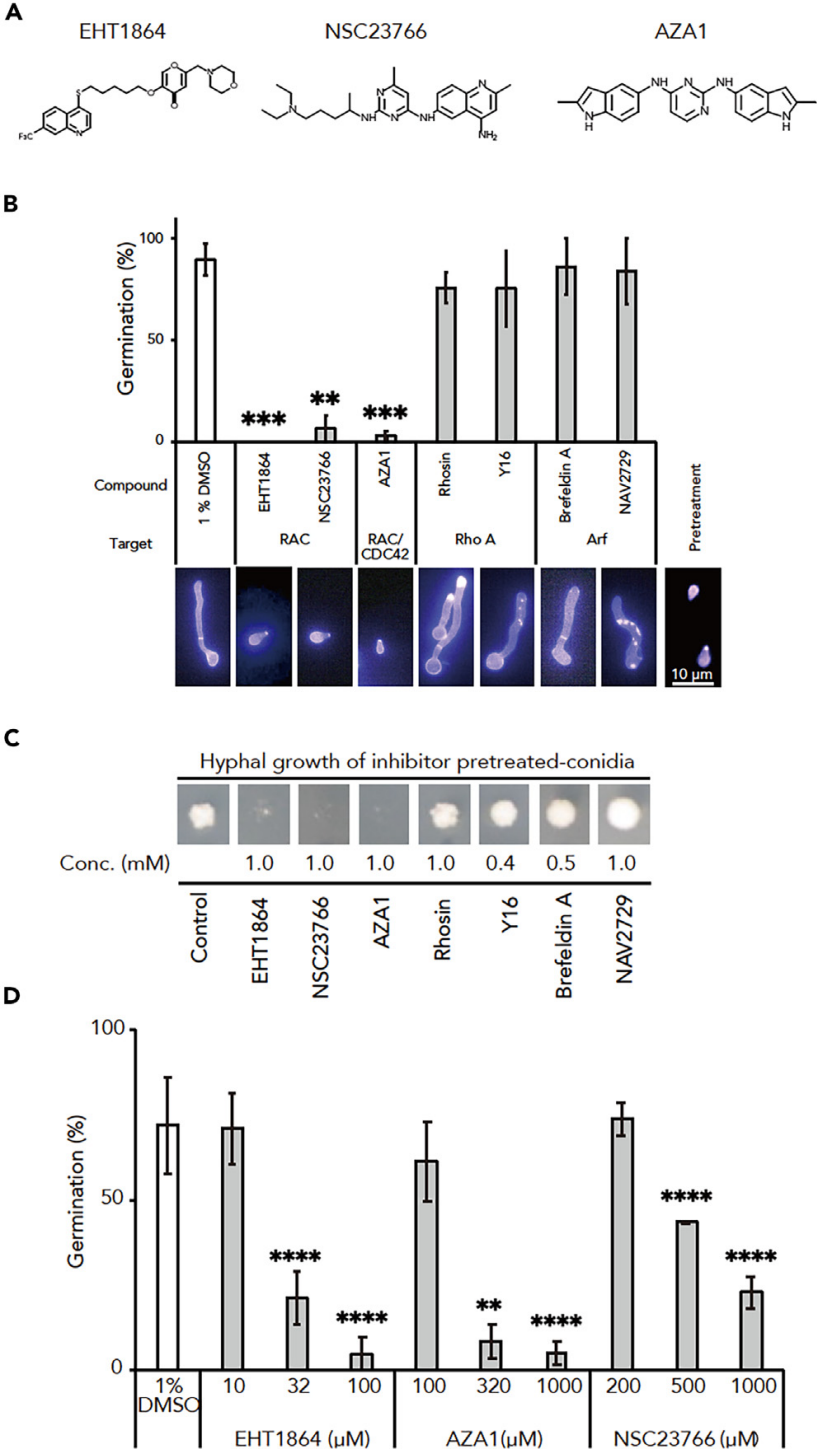
Mammalian Rac and Rac/Cdc42 inhibitors suppress conidial germination in *Trichophyton rubrum* (A) Structures of Rac inhibitors EHT1864 and NSC23766 and Rac/Cdc42 inhibitor AZA1. (B) Effects of mammalian small GTPase inhibitors on conidial germination in *T. rubrum* were observed. Conidia were stained with calcofluor white. *n* = 3 each. ∗∗, *p* < 0.01; ∗∗∗, *p* < 0.001. Mean ± SD. The lower panel showed representative fungal cell. (C) Effects of mammalian small GTPase inhibitors on mycelial growth in *T. rubrum* were observed. (D) Effects of mammalian Rac and Rac/Cdc42 inhibitors EHT1864, AZA1, and NSC23766 on conidial germination in *T. rubrum* were observed. Conidia were stained with calcofluor white. *n* = 3 each. ∗∗, *p* < 0.01; ∗∗∗∗, *p* < 0.0001. Mean ± SD.

To investigate a small GTPase pathway involved in conidial germination and mycelial growth in *T. rubrum*, we screened small GTPase inhibitors *in cellulo* by culturing fungal conidia with those inhibitors. Seven compounds known to inhibit mammalian Rho and Arf family proteins—Rac (EHT1864 and NSC23766), Rac and Cdc42 (AZA1), Rho A (Rhosin and Y16), and Arf (Brefeldin A and NAV2729)—were used.^33^^,^^34^^,^^35^^,^^36^^,^^37^^,^^38^ For Rac inhibitors, we used EHT1864 and NSC23766 as these are well-established in mammals^33^^,^^34^^,^^39^^,^^40^^,^^41^^,^^42^; EHT1864, NSC23766, and AZA1 (Figure 1A) hindered conidial germination and mycelial growth in *T. rubrum*, whereas Rhosin, Y16, Brefeldin A, and NAV2729 did not (Figures 1B and 1C). These Rac and Cdc42 inhibitors suppressed conidial germination in a dose-dependent manner (Figure 1D). The IC_50_ of EHT1864, NSC23766, and AZA1 were 16, 640, and 127 μM, respectively (Table 1). These findings imply that Rac and/or Cdc42 signaling is crucial for conidial germination and hyphal growth in *T. rubrum*, suggesting these pathways as potential therapeutic targets for dermatophytosis.

**Table 1:** Effect of mammalian Rac and/or Cdc42 inhibitors against TrCdc42, TrRac, and conidial germination in *T. rubrum*

Inhibitor	Target in mammalian cells	TrCdc42 IC_50_ (μM)	TrRac IC_50_ (μM)	IC_50_ to *T. rubrum* conidial germination (μM)
AZA1	Rac1 and Cdc42	147	127	127
CASIN	Intersetin1/Cdc42	192	518	20
EHop-016	Vav/Rac	40	137	45
EHT1864	Rac1	154	>1,000	16
ITX3	TrioN/Rac, Rho	>50	>50	>50
Ketorolac	Rac1, Cdc42	889	1,416	363
MBQ-167	Rac, Cdc42	>100	>100	>100
ML-141	Cdc42	>1,000	>1,000	1.7
NSC23766	Tiam-1, Trio/Rac	368	477	640
ZCL278	Intersetin1/Cdc42	>1,000	>1,000	>1,000

### XP_003237709 and XP_003234353 are homologs of Rac and Cdc42 in *T. rubrum*, respectively

**Figure 2:**
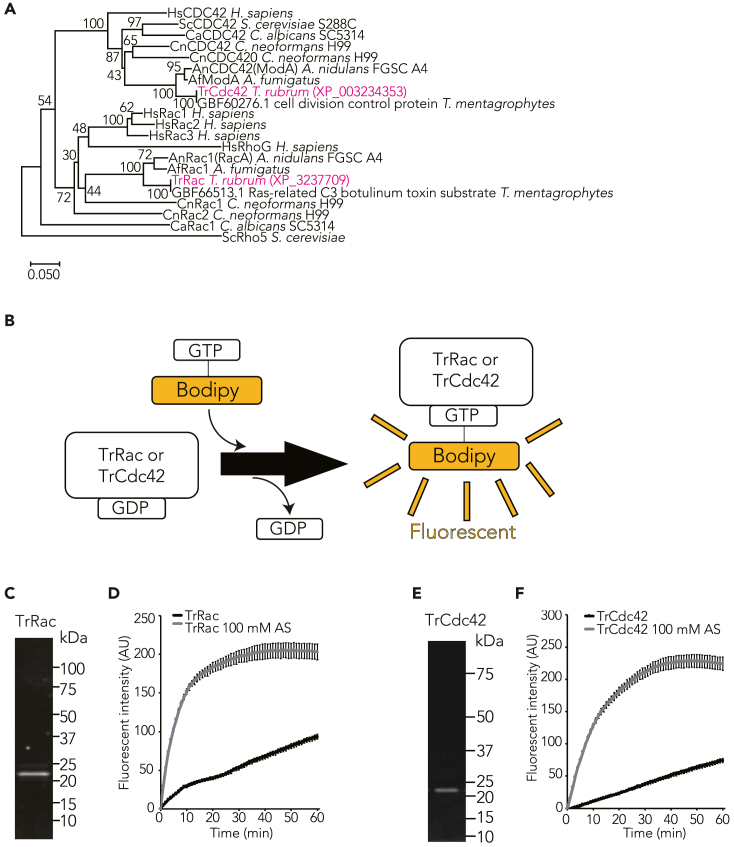
XP_003237709.1 and XP_003234353.1 are TrRac and TrCdc42 in *T. rubrum* (A) Phylogenetic tree of fungal Rac and Cdc42 proteins, as prepared using the neighbor-joining method. The percentage of replicate trees clustering taxa together in bootstrap test (500 replicates) is indicated at branches. Evolutionary distances were calculated using Poisson correction method in terms of amino acid substitutions per site. (B) Fluorescence-based guanine nucleotide exchange assay method. (C) SDS-PAGE analysis of purified recombinant TrRac proteins stained with stain-free fluorescence stain. (D) TrRac guanine nucleotide exchange assay was performed. 1 μM recombinant TrRac and 0.1 μM bodipy GTP were mixed with or without 100 mM ammonium sulfate (AS), and fluorescence intensity was measured. Mean ± SD. (E) SDS-PAGE analysis of purified recombinant TrCdc42-His proteins stained with stain-free fluorescence stain. (F) TrCdc42-His guanine nucleotide exchange assay was performed. 1 μM recombinant TrCdc42-His and 0.1 μM bodipy GTP were mixed with or without 100 mM AS, and fluorescence intensity was measured. Mean ± SD.

Rac and Rac/Cdc42 inhibitors fall into two categories: one inhibiting the binding of Rac/Cdc42 to guanine nucleotides, e.g., EHT1864,^40^ and the other inhibiting the binding of Rac/Cdc42 to their GEF, e.g., NSC23766.^35^ As both types of inhibitors inhibited hyphal growth of *T. rubrum* (Figure 1), we focused on identifying Rac and Cdc42 homologs in *T. rubrum*. Through a BLAST search in the genome sequence,^43^ we identified XP_003237709 and XP_003234353 of *T. rubrum* CBS118892 as candidate homologs of human Rac1 (identity: 80%) and Cdc42 (identity: 78%). Phylogenetic tree analysis demonstrated that XP_003237709 and XP_003234353 were related to Rac and Cdc42 proteins, respectively, from other fungal species *S. cerevisiae*, *C. albicans*, *C. neoformans*, *A. nidulans*, *Aspergillus fumigatus*, and *Trichophyton mentagrophytes* (Figure 2A). We purified recombinant XP_003237709 and hexahistidine (His)-tagged XP_003234353 proteins expressed in *E. coli* (Figures 2C and 2E). Ammonium sulfate accelerates guanine nucleotide exchange of GTPases.^44^ These proteins bound to bodipy-labeled GTP in an ammonium sulfate-dependent manner (Figures 2B–2D and 2F), indicating that XP_003237709 and XP_003234353 are guanine nucleotide-binding proteins. These findings affirm that XP_003237709 and XP_003234353 are homologs of Rac and Cdc42 in *T. rubrum* and are subsequently referred to as TrRac and TrCdc42, respectively.

### *T. rubrum* Cdc24 promotes guanine nucleotide exchange of TrRac and TrCdc42

**Figure 3:**
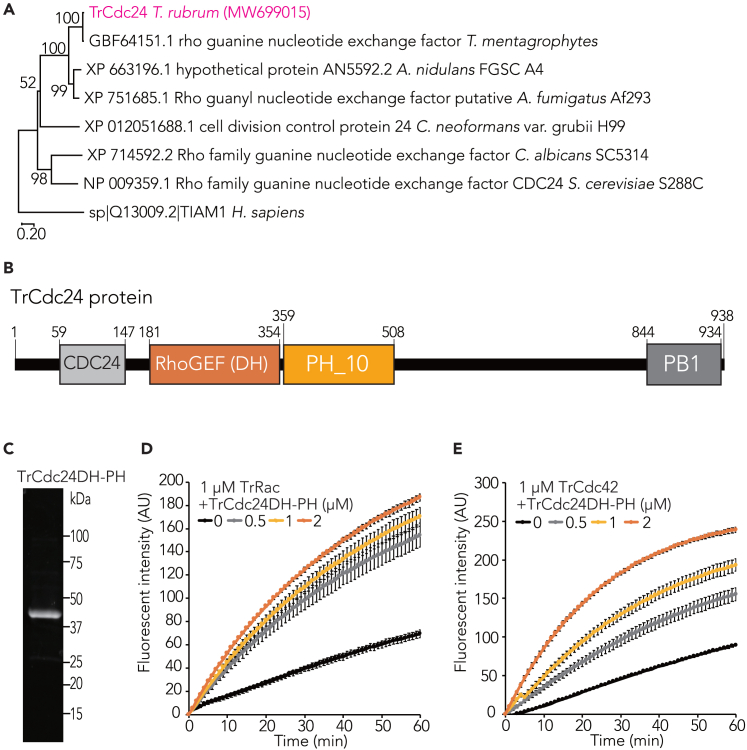
*T. rubrum* Cdc24 stimulates guanine nucleotide exchange of Rac and Cdc42 (A) Phylogenetic tree analysis of fungal Cdc24 and human Tiam1 using the neighbor-joining method. The percentage of replicate trees clustering taxa together in bootstrap test (500 replicates) is indicated at branches. Evolutionary distances were calculated using Poisson correction method in terms of amino acid substitutions per site. (B) Protein domains of Cdc24 shown in an image. (C) SDS-PAGE analysis of purified recombinant DH-PH domain of TrCdc24 proteins stained with stain-free fluorescent stain. (D) Guanine nucleotide exchange of TrRac with or without TrCdc24DH-PH was observed. 1 μM TrRac and 0.1 μM bodipy GTP were incubated with or without 0.5, 1, and 2 μM TrCdc24DH-PH. Mean ± SD. (E) Guanine nucleotide exchange of TrCdc42-His with or without TrCdc24DH-PH was observed. 1 μM TrCdc42-His and 0.1 μM bodipy GTP were incubated with or without 0.5, 1, and 2 μM TrCdc24DH-PH. Mean ± SD.

In the filamentous fungi *N. crassa* and *C. purpurea*, DH-PH domain-containing proteins act as GEFs, i.e., these proteins promote the conversion of Rho-type GTPases, such as Rac and Cdc42, from GDP-bound to GTP-bound forms.^28^^,^^29^ We identified XP_003232406 as a DH-PH domain-containing protein in the genome of *T. rubrum*. We cloned this cDNA, but the size of the PCR product (registered in GenBank as MW699015) was 402 bp shorter than the predicted size from XP_003232406. Sequence alignment analysis revealed that the last part of the third exon of XP_003232406.1 was missing in MW699015 (Figure S1A). As this sequence was also missing in Cdc24 cDNAs from other filamentous fungi (Figure S1B), we assumed that this additional sequence in XP_003232406 is due to misprediction of the splice site. The sequence similarity of the protein encoded by MW699015 to *S. cerevisiae* Cdc24 was 27%. Phylogenetic tree analysis revealed that MW699015 and Cdc24 proteins from other fungi belonged to the same clade (Figure 3A). We therefore designated the protein encoded by MW699015 as TrCdc24. A purified recombinant peptide containing DH-PH domains (from 150^th^ to 508^th^ amino acids in Figure 3B) expressed in *E. coli* (Figures 3B and 3C) enhanced the guanine nucleotide exchanges of TrRac and TrCdc42-His (Figures 3D and 3E). These results suggest that TrCdc24 is a GEF for TrRac and TrCdc42.

### Repression of *Trcdc24* leads to inhibition of mycelial growth

**Figure 4:**
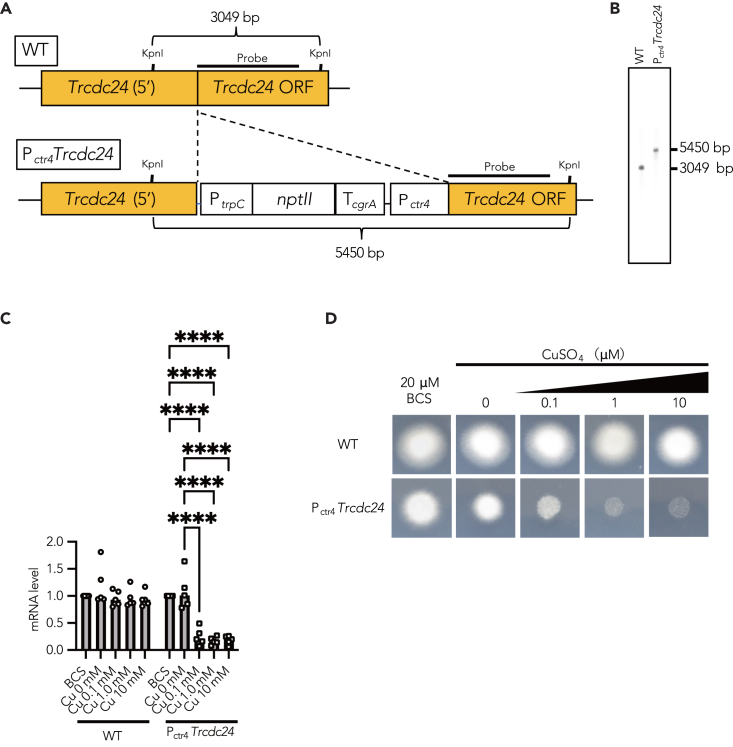
Repression of *Trcdc24* shows mycelial growth inhibition (A) Schematic representation of the *Trcdc24* locus in the genome of *T. rubrum* CBS118892 WT and P_*ctr4*_*Trcdc24* mutant. (B) Southern blot analysis of genome DNA samples from *T. rubrum* CBS118892 WT and P_*ctr4*_*Trcdc24*. (C) Quantification of *Trcdc24* mRNA in total RNA of *T. rubrum* CBS118892 and P_*ctr4*_*Trcdc24* cultured in medium containing 0, 0.1, 1, or 10 μM CuSO_4_ or 20 μM BCS, a copper ion chelator. *n* = 5 each. ∗∗∗∗, *p* < 0.0001. Mean ± SD. (D) Mycelial growth of *T. rubrum* CBS 118892 and P _*ctr4*_*Trcdc24* on an agar plate with 0, 0.1, 1, or 10 μM CuSO_4_ or 20 μM BCS.

As homokaryotic knockout of Cdc24 is lethal in *N. crassa*,^28^ it is anticipated that TrCdc24 is similarly indispensable for the survival of *T. rubrum*. To circumvent the potential lethality associated with TrCdc24 knockout, we sought to establish a conditional gene repression system. We used a copper-responsive promoter (P_*ctr4*_), a system employed in another dermatophyte, *T*. *mentagrophytes* (formerly *Arthroderma vanbreuseghemii*), to repress gene expression in a copper concentration-dependent manner.^45^ We integrated P_*ctr4*_ upstream of the open reading frame of *Trcdc24* (Figure 4A) and confirmed its integration through Southern blot analysis (Figure 4B). The expression level of *Trcdc24* mRNA was significantly reduced in P_*ctr4*_*Trcdc24* mutant *T. rubrum* cultured with copper ions, depending on the copper ion concentration, compared with that in wild type (WT) and P_*ctr4*_*Trcdc24* mutant *T. rubrum* cultured with the copper chelator bathocuproinedisulfonic acid (Figure 4C). This copper ion concentration-dependent decrease in *Trcdc24* mRNA expression was not observed in WT (Figure 4C). These results suggest that the P_*ctr4*_ system functions effectively in *T. rubrum*. Intriguingly, the P_*ctr4*_*Tr*c*dc24* mutant experienced arrested mycelial growth under gene silencing conditions in a copper ion concentration-dependent manner (Figure 4D), highlighting the essential role of TrCdc24 in the mycelial growth of *T. rubrum*.

### TrCdc42 is essential for mycelial growth in a TrRac deletion background

**Figure 5:**
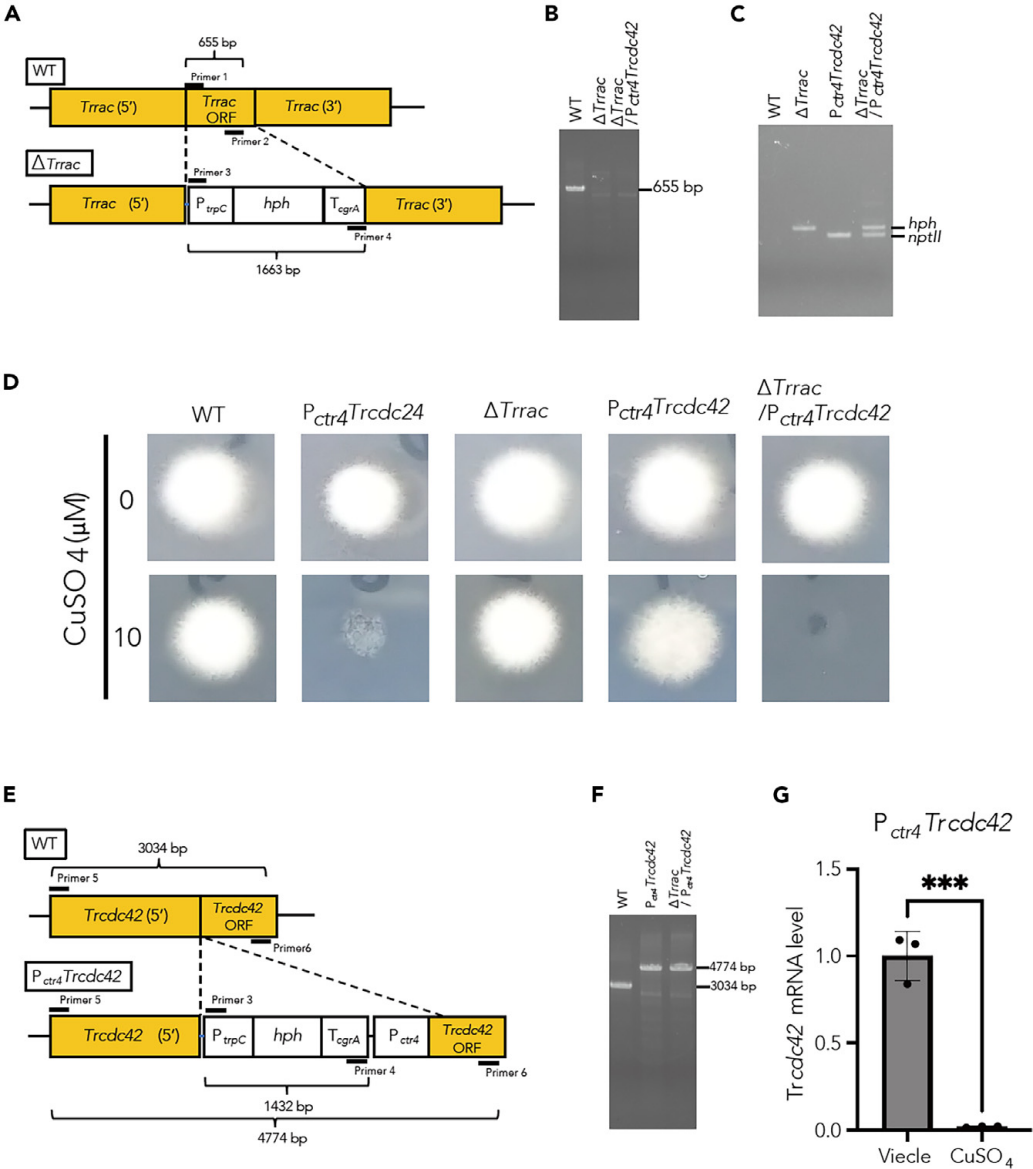
Repression of *Trcdc42* shows mycelial growth inhibition in a TrRac deletion background (A) Schematic representation of the *Trrac* locus in the genome of *T. rubrum* CBS118892 WT and *Trrac* deletion mutant (Δ*Trrac*). (B) PCR analysis conducted on genomic DNA samples from *T. rubrum* CBS118892 WT, P_*ctr4*_*Trrac*, and Δ*Trrac*/P _*ctr4*_*Trcdc42* using primers 1 and 2 as depicted in A. (C) PCR analysis was conducted on genomic DNA samples from *T. rubrum* CBS118892 WT, P_*ctr4*_*Trrac*, P _*ctr4*_*Trcdc42*, and Δ*Trrac*/P _*ctr4*_*Trcdc42* using primers 3 and 4 as depicted in A. (D) Mycelial growth of *T. rubrum* CBS 118892, P _*ctr4*_*Trcdc24*, Δ*Trrac*, P _*ctr4*_*Trcdc42*, Δ*Trrac*/P _*ctr4*_*Trcdc42* on an agar plate with 0 or 10 μM CuSO_4_. (E) Schematic representation of the *Trcdc42* locus in the genome of *T. rubrum* CBS118892 WT and P_*ctr4*_*Trcdc42* mutant. (F) PCR analysis was conducted on genomic DNA samples from *T. rubrum* CBS118892 WT, P _*ctr4*_*Trcdc42*, and Δ*Trrac*/P _*ctr4*_*Trcdc42* using primers 5 and 6 as depicted in A. (G) Quantification of *Trcdc42* mRNA in total RNA of *T. rubrum* CBS 118892 and P_*ctr4*_*Trcdc42* cultured in medium containing 0 or 10 μM CuSO_4_. *n* = 3 each. ∗∗∗, *p* < 0.001. Mean ± SD.

Since mammalian Rac inhibitors inhibited conidial germination and hyphal formation in *T. rubrum* (Figures 1B–1D) and repression of the TrRac GEF TrCdc24 caused a defect in mycelial growth (Figure 4D), we generated a conditional *Trrac* suppression mutant (P_*ctr4*_*Trrac*) and observed mycelial growth. Contrary to our expectation, no difference in mycelial growth was observed between the fungi under induced and suppressed gene expression conditions in the P_*ctr4*_*Trrac* mutant (data not shown). Furthermore, we were able to knock out *Trrac*. *Trrac* deletion and selection-marker insertion in this strain were confirmed through PCR (Figures 5A–5C). Furthermore, the mRNA level of *Trrac* in the Δ*Trrac* strain was confirmed to be reduced to a level comparable to the limit of detection (data not shown). No apparent defect in mycelial growth was observed in the strain obtained (Figure 5D). Since the amino acid sequence of TrCdc42 was similar to that of TrRac and the guanine nucleotides bound to TrCdc42 were exchanged by TrCdc24 as to TrRac, we hypothesized that TrCdc42 acts in concert with TrRac in promoting mycelial growth. Therefore, we investigated the inhibitory effect of Rac inhibitors on the guanine nucleotide exchange reaction of TrCdc42 by TrCdc24. TrCdc42 guanine nucleotide exchange by TrCdc24 was inhibited by the Rac inhibitors EHT1864 and NSC23766 with IC_50_ of 154 and 368 μM, respectively (Table 1). To further investigate the function of TrCdc42 in mycelial growth, we generated a conditional *Trcdc42* repression mutant (P_*ctr4*_*Trcdc42*) and a Δ*Trrac* and P_*ctr4*_*Trcdc42* double mutant and confirmed the insertion of the fragment, which contained selective marker and *ctr4* promoter, into targeted region through PCR (Figures 5E and 5F). In P_*ctr4*_*Trcdc42* strain, *Trcdc42* mRNA level was decreased under CuSO_4_ treatment (Figure 5G). P_*ctr4*_*Trcdc42* showed a slight growth defect under repressive conditions, whereas this growth defect was enhanced in the *Trrac*-deficient/P_*ctr4*_*Trcdc42* strain under repressive conditions (Figure 5D). These results suggest that TrRac and TrCdc42 have similar essential roles in mycelial growth and that the TrCdc24-TrCdc42/TrRac pathway is essential for mycelial growth.

### Repression of TrCdc24 leads to abnormal cell morphology, actin delocalization, and cell death

**Figure 6:**
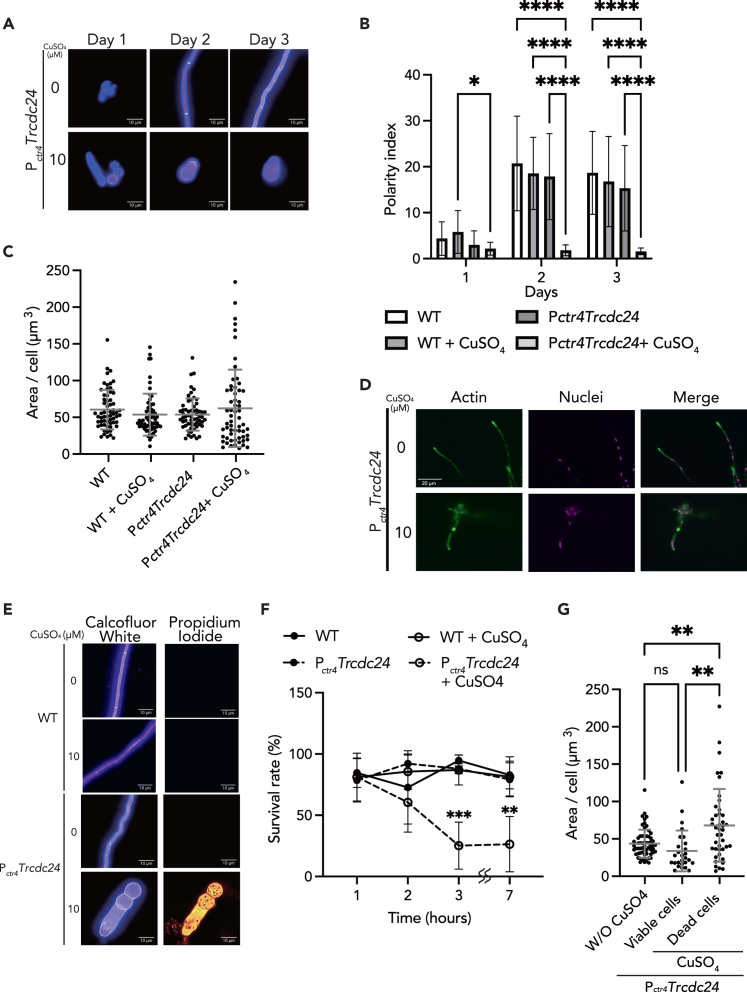
Repression of *Trcdc24* shows abnormal cell formation, actin localization, cell death, and reduced cell viability (A) Morphology of P_*ctr4*_*Trcdc24* cells was observed for 3 days under *cdc24* repression condition (with 10 μM CuSO_4_) or non-repression condition (without CuSO_4_). Fungal cells were stained with calcofluor white before observation. (B) Polarity of WT and P_*ctr4*_*Trcdc24* cells cultured with or without 10 μM CuSO_4_. Fungal cells were stained with calcofluor white. The polarity index was calculated on day 1 (*n* = 50–60 each), day 2 (*n* = 50–60 each), and day 3 (*n* = 60 each). ∗, *p* < 0.05; ∗∗∗∗, *p* < 0.0001. Mean ± SD. (C) Cell area was measured on day 3. The dots on the graph represent the value of the individual cell area. *n* = 60 each. Mean ± SD. (D) Actin (green) and DNA (magenta) were visualized under *Trcdc24* repression condition (with 10 μM CuSO_4_) or non-repression condition (without CuSO_4_) in Pctr4*Trcdc24* cells. (E) WT and P_*ctr4*_*Trcdc24* cells were stained with calcofluor white and PI. (F) The survival rate of WT and P_*ctr4*_*Trcdc24* cells was observed. *n* = 3 each. ∗∗, *p* < 0.01; ∗∗∗, *p* < 0.001. Mean ± SD. (G) The area of P_*ctr4*_*Trcdc24* cells was measured on day 7. The dots on the graph represent the value of the individual cell area. The numbers of W/O CuSO_4_, viable, and dead cells were 60, 27, and 42, respectively. ∗∗, *p* < 0.01. Mean ± SD.

To gain insights into the halted mycelial growth in *T. rubrum* when the TrCdc24-Tr Cdc42/TrRac pathway is repressed, we conducted a chronological observation of the morphology of P_*ctr4*_*Trcdc24* (Figure 6A). In the absence of copper ions, oval cells observed on day 1 elongated into filamentous cells on day 2. This morphological elongation between culture days 1 and 2 was evident while calculating the polarity index (major axis length/minor axis length) (Figure 6B). On the contrary, the P_*ctr4*_*Trcdc24* mutant cultured with copper ions for 2 or 3 days exhibited a more rounded morphology, characterized by a shorter major axis and a longer minor axis compared with the mutant cells cultured without copper ions (Figure 6A). The polarity index of the copper-treated cells was significantly lower than that of the untreated cells (Figure 6B). Interestingly, by day 3, the size of the copper-treated cells showed no significant difference from that of the untreated cells (Figure 6C). These findings indicate that hyphal formation is regulated by TrCdc24.

Both Rac and Cdc42 play roles in facilitating actin organization.^21^ In filamentous fungi, F-actin localizes to the hyphal tip and forms higher-order structures known as actin patches and actin cables.^46^ These structures are responsible for endocytosis and polarized transport, respectively. In the absence of copper ions, actin was localized at the hyphal tip (Figure 6D). Conversely, *Trcdc24* repression impaired actin localization at the hyphal tip, resulting in abnormal actin accumulation in the cytosol (Figure 6D). This leads to the conclusion that TrCdc24 regulates the localization of actin at the hyphal tip.

In yeast, actin dysfunction leads to cell death.^47^ To elucidate whether TrCdc24 functions in cell death, dead cells were stained with propidium iodide (PI), a dye permeating dead cells but not living cells. Under the repression condition, P_*ctr4*_*Trcdc24* mutants were stained with PI after culturing for 3 days (Figure 6E). The survival rate of *Trcdc24*-repressed cells was reduced to 22% after 3 days of culture (Figure 6F). To further analyze the relationship between abnormalities in actin-regulated cell morphology and cell death, we compared the size of living and dead cells under *Trcdc24*-suppressed conditions. The results showed an increase in cell size in dead cells (Figure 6G). These findings suggest that TrCdc24 contributes to mycelial growth by modulating cell morphology and viability.

### EHop-016, a TrCdc24 inhibitor, ameliorates *in vitro* nail infection and lethality in an invertebrate infection model of dermatophytosis

**Figure 7:**
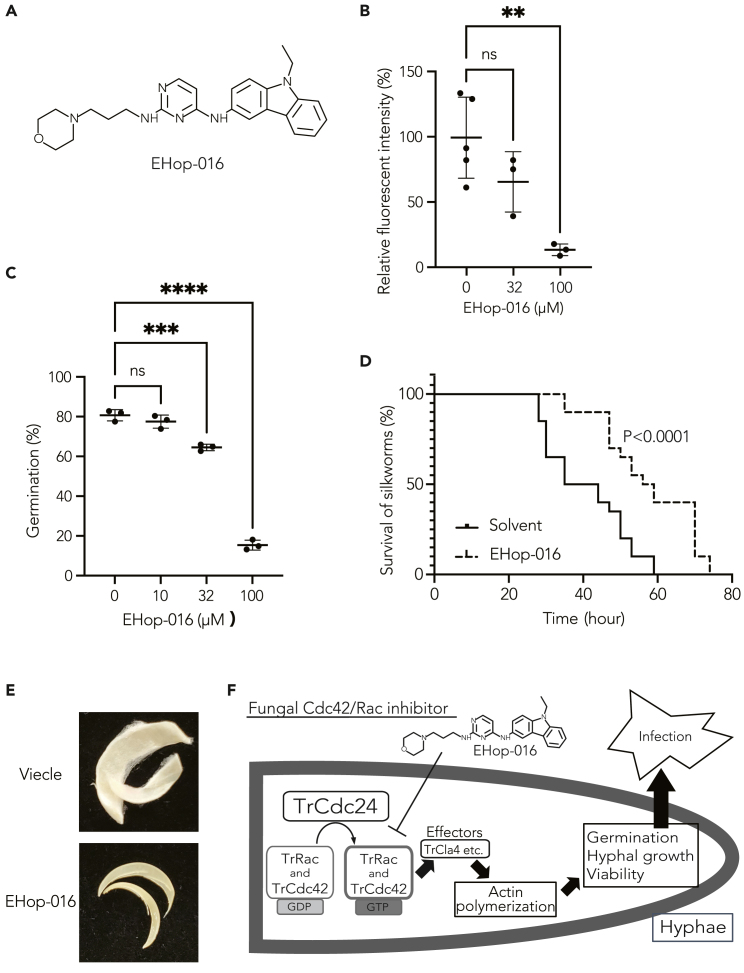
Therapeutic efficacy of EHop-016 against an invertebrate infection model of dermatophytosis (A) Structure of the fungal Cdc42 and Rac inhibitor EHop-016. (B) Inhibitory effect of EHop-016 on guanine nucleotide exchange of TrCdc42-His by TrCdc24DH-PH. 0 (*n* = 5), 32 (*n* = 3), and 100 (*n* = 3) μM of EHop-016 were added. ∗∗, *p* < 0.01. Mean ± SD. (C) Inhibitory effect of EHop-016 on conidial germination was observed. *n* = 3 each. ∗∗∗, *p* < 0.001; ∗∗∗∗, *p* < 0.0001. Mean ± SD. (D) Therapeutic effect of EHop-016 in a silkworm infection assay with *T. rubrum*. (E) Inhibitory effect of EHop-016 on the growth of *T. rubrum* on human nails. After 36 days of incubation at 28°C, the nail fragments were observed. On the solvent-treated nail (Viecle), a fungal lawn was observed, but no fungal lawn was observed on the EHop-016-treated nail. (F) Model. TrCdc42 and TrRac are activated to their GTP-bound forms by TrCdc24. Subsequently, the activated GTPases bind to effector molecules, like TrCla4, to enhance the polymerization of actin. This, in turn, promotes conidial germination, hyphal growth, and viability, ultimately leading to an enhanced infection by the fungus. The employment of a fungal Cdc42 and Rac inhibitor, EHop-016, demonstrated the potential to mitigate the fungal infection.

Given the implications from the aforementioned results, suggesting that the activation mechanism of TrRac and TrCdc42 is a promising target for antifungal drug development, we screened Rac and Cdc42 inhibitors previously reported in mammals, including EHop-016, AZA1, EHT1864, CASIN, NSC23766, ketorolac, MBQ-167, ML-141, ZCL278, and ITX3.^48^ Employing an *in vitro* biochemical assay, we evaluated the impact of Rac/Cdc42 inhibitors on the potentiating effect of TrCdc24 in the GDP/GTP exchange reaction of TrRac and TrCdc42 (Table 1). Notably, the results revealed that EHop-016 exhibited the strongest activity against TrCdc42, with an IC_50_ of 40 μM (Figures 7A and 7B; Table 1). Inhibitors showing TrCdc42 inhibitory activity in the biochemical assay also inhibited conidial germination *in cellulo* (Figure 7C; Table 1). Intriguingly, the IC_50_ of these inhibitors against TrCdc42 showed a higher correlation coefficient with fungal conidial germination inhibition than that of inhibitors against TrRac (0.58 and 0.39, respectively). These findings suggest that TrCdc42 plays a more crucial role than TrRac in *T. rubrum* conidial germination, consistent with the observations that the P_*ctr4*_*Trcdc42* strain exhibited partially reduced mycelial growth under gene repression conditions, while the P_*ctr4*_TrRac and Rac deletion strains displayed no change in mycelial growth under all tested conditions (Figure 5A).

Given that *T. rubrum* is an anthropophilic but not zoophilic dermatophyte, establishing an *in vivo* infection model in vertebrates is challenging.^49^ To determine the efficacy of chemical inhibition of the Cdc42/Rac pathway in animal infection, we used silkworms, a well-established invertebrate infection model for various fungi, including dermatophytes.^48^^,^^49^^,^^50^ EHop-016 was selected as the test compound due to its higher TrCdc42 inhibitory activity, and its pharmacological parameters have already been reported in mice.^50^ EHop-016 significantly extended the survival of animals infected with dermatophytes (Figure 7D), indicating that an inhibitor targeting the Cdc42/Rac pathway assists animals in recovering from dermatophyte infection.

Onychomycosis, primarily caused by *T. rubrum*, represents a challenging and recurring nail infection.^51^ To elucidate the effect of the Cdc42/Rac inhibitor against *T. rubrum* nail infection, we tested EHop-016 in an *in vitro T. rubrum* nail infection model. Treatment with 2.6% EHop-016 resulted in lesser *T. rubrum* growth on the nails compared with the vehicle treatment control (Figure 7E), confirming that the Cdc42/Rac inhibitor EHop-016 inhibits *T. rubrum* hyphal growth on nails.

## Discussion

In the realm of antifungal chemotherapy, significant challenges arise due to the limited pool of target molecules and the emergence of chemoresistant fungi against existing drugs.^52^ Here, through the development of a gene silencing system and chemical screening in *T. rubrum*, we made two pivotal discoveries. First, we identified the TrCdc24-TrCdc42/TrRac pathway as a promising therapeutic target for dermatophytosis. Second, we identified chemical inhibitors that target the TrCdc24-TrCdc42/TrRac pathway. Our study demonstrated that TrCdc24 serves as a GEF for TrCdc42/TrRac and is indispensable for mycelial growth, cell morphogenesis, and viability. Inhibitors of Cdc42/Rac curtail the activation of TrCdc42 and TrRac by TrCdc24, subsequently impeding the conidial germination of *T. rubrum*. EHop-016, the most potent Cdc42 inhibitor in this study, showcased the potential to ameliorate the lethality observed in silkworms injected with *T. rubrum*, a well-established *in vivo* model for dermatophyte infection.^53^ This study underscores the possibility that inhibiting the TrCdc24-TrCdc42/TrRac signaling pathway could serve as an effective therapeutic strategy against fungal infections.

### Function of the TrCdc24-TrCdc42/TrRac pathway

In both animals and fungi, Cdc42 and Rac play pivotal roles in actin organization.^21^ The present study demonstrates that TrCdc24 enhances the guanine nucleotide exchange reaction of TrCdc42 and TrRac (Figure 3) and reveals that repression of TrCdc24 expression impacts hyphal formation, actin localization at the hyphal tip, and cell viability. Biochemical, genetic, and chemical-biological analyses suggested that TrCdc42 and TrRac proteins also contribute to mycelial growth in *T. rubrum*. Furthermore, the regulation of actin localization by TrCdc24 is likely mediated by the activation of TrCdc42 and TrRac. Recently, we reported that TrCla4 is a p21-activated kinase (PAK) in *T. rubrum* that is activated by TrRac and may regulate mycelial growth by enhancing actin polymerization.^54^ The TrCdc24-TrCdc42/TrRac-TrCla4 pathway may promote hyphal growth by regulating actin dynamics (Figure 7F). While the downstream signaling of TrCdc42 and TrRac is not fully clarified, studies on other filamentous fungi and yeast suggest that the downstream signal may be mediated not only by PAKs but also by effector molecules that regulate actin polymerization, such as formin.^26^^,^^55^ In a yeast actin mutant strain with reduced ATPase activity, cell death was observed along with abnormal localization of actin filament and subsequent loss of actin filament.^56^ Similarly, cell death was observed in the TrCdc24-suppressed strain with abnormal actin localization, suggesting that control of actin localization may also contribute to the survival of dermatophytes.

### Potential of antifungal drug discovery by targeting Cdc42 and Rac: Its generality and specificity

Cdc42 and Rac play a significant role in cell migration and defense against external stimuli in human skin.^57^^,^^58^^,^^59^ Furthermore, inhibition of Pak2, an effector of Cdc42 and Rac, may lead to acute cardiovascular toxicity.^60^ Thus, the use of TrCdc42 and TrRac inhibitors for the treatment of dermatophytosis could potentially cause adverse effects, such as immunosuppression, infections, and acute cardiovascular toxicity, through the inhibition of human Cdc42, Rac, and/or off-targets. These side effects could be avoided due to the low amino acid sequence identity (approximately 27%) between human Rho GEFs and *T. rubrum* Cdc24. Therefore, the Cdc24-mediated Cdc42/Rac activation machinery is a promising target for antifungal drug development. The conidial germination inhibition assay revealed that MBQ-167, a potent mammalian Rac and Cdc42 GEF interaction inhibitor, did not show any inhibitory activity against the fungus even at 100 μM.^61^ This suggests that the compounds can recognize differences between mammalian and fungal p21 (Rac and Cdc42). Calcineurin, another signaling molecule that controls hyphal growth, has been proposed as a target molecule for antifungal drug development.^62^ Although FK506 (tacrolimus), an approved immunosuppressant and inhibitor of calcineurin, inhibits fungal growth *in vitro*,^63^ its immunosuppressive effect is a deterrent for antifungal drug development. To overcome this problem, derivatization has been attempted, and some derivatives have successfully reduced the immunosuppressive action despite having strong antifungal activity.^63^^,^^64^ In addition, the crystal structure of the calcineurin-FK506-FKBP12 complex was recently elucidated and used for the design of novel fungal-specific FK506 analogs.^65^ Structural analysis and derivatization of lead compounds such as EHop-016 are expected to be powerful strategies for the discovery of fungal-selective Cdc42/Rac inhibitors.

The amino acid sequences of the Cdc24 GEF exhibit striking similarity among filamentous fungi, with a notable 73% identity to the DH-PH domain of *A*. *fumigatus* Cdc24. Cdc42 and/or Rac play pivotal roles in hyphal growth in pathogenic fungi, such as *A. niger* and *C. albicans*, in which Cdc24 is an indispensable protein for hyphal growth.^26^^,^^32^ Considering these findings, it is highly plausible that inhibitors targeting the fungal Cdc24-Cdc42/Rac pathway can serve as broad-spectrum antifungal drugs. In future studies, it would be interesting to investigate the effects of these compounds on pathogenic fungi, including *C. albicans*, *A*. *fumigatus*, and *C. neoformans*, in terms of the adaptable range of this target. These findings of the present study provide information on the extent of the antifungal spectrum and the potential of targeting the fungal Cdc24-Cdc42/Rac pathway for further applications.

### Difference between animal and fungal targets of Cdc42 and Rac inhibitors

Cdc42 and Rac have garnered attention as potential anticancer drug targets, leading to the discovery of over 30 mammalian Cdc42 and/or Rac inhibitors.^48^ Our chemical inhibitor screening revealed a notable difference in selectivity between mammalian and fungal Cdc42/Rac. In particular, mammalian Rac-selective inhibitors, EHT1864, and EHop-016 showed higher selectivity for Cdc42 than for Rac in fungi (Table 1). This finding challenges the conventional notion that mammalian Rac inhibitors selectively inhibit fungal Cdc42 and Rac, akin to their selectivity in mammals. The mechanism underlying this selectivity of inhibitors remains unclear, and this discovery provides valuable insights for understanding the function of fungal Cdc42 and Rac using these inhibitors.

In the present study, we identified EHop-016 as a fungal Cdc42/Rac inhibitor. As this compound also inhibits mammalian Rac,^66^ it is difficult to use EHop-016 directly as a therapeutic agent. EHop-016 could serve as a useful tool to better understand the function of the Cdc24-Cdc42/Rac pathway in fungi.

### A genetic tool to analyze genes of interest

*T. rubrum* is the most common causative fungus of dermatophytosis;^1^ however, the molecular mechanism governing its hyphal growth remains unclear. A prior histological study suggested that hyphal growth is essential for *T. rubrum* virulence,^12^ but limited genetic tools and the slow growth of this fungus have prevented further analysis. Several studies have reported on the genetic modification of this fungus.^14^^,^^15^^,^^17^^,^^67^^,^^68^ Recently, auxotrophic mutants of *T. rubrum* have been generated using the Cas9 system.^69^ In these studies, gene deletions and insertions were performed for functional analysis. Previous methods have made it difficult to analyze the functions of essential proteins that are potential drug targets. In the present study, we implemented a copper-responsive promoter system previously used in *Arthroderma vanbreuseghemii*,^45^^,^^70^ enabling us to explore essential gene functions and exercise spatiotemporal regulation of gene expression. This system is poised to streamline the investigation of *T. rubrum* pathogenicity and identification of novel drug targets.

### Conclusion

We herein demonstrated that the chemical and genetic inhibition of the TrCdc42/TrRac pathway leads to growth arrest in *T. rubrum*. We also proposed an approach that combines genetic and biochemical methods to discover novel antifungal compounds against *T. rubrum*. It is anticipated that screening for more potent and selective inhibitors of the fungal Cdc24-Cdc42/Rac pathway will yield novel antifungal agents. This study presents Cdc42-Cdc42/Rac signaling as a novel drug target, distinct from those of existing antifungal drugs.

### Limitations of the study

This study showed that EHop-016 inhibits conidial germination, growth on nails, and silkworm-killing ability in *T. rubrum* through inhibition of the Cdc24-Cdc42/Rac pathway. Given that small-molecule inhibitors possess off-target effects in addition to their intended targets,^71^^,^^72^ EHop-016 may exert these activities by inhibiting pathways other than the fungal Cdc24-Cdc42/Rac pathway. In particular, compounds that competitively inhibit the GTP binding of small GTPases may also affect the guanine nucleotide-binding sites of other GTP-binding proteins. The development of inhibitors for the fungal Cdc24-Cdc42/Rac pathway may involve searching for compounds that inhibit the interaction between Cdc24 and Cdc42/Rac rather than the guanine nucleotide-binding site, which would lead to more specific inhibitors.

## STAR★Methods

### Key resources table

**Table:** 

REAGENT or RESOURCE	SOURCE	IDENTIFIER
**Antibodies**		

Anti-β-Actin Antibody (C4)	Santa Cruz Biotechnology	Cat# sc-47778; RRID:AB_626632
Donkey Anti-Mouse IgG H&L (Alexa Fluor® 488) preadsorbed	Abcam	Cat# ab150109; RRID:AB_2571721

**Bacterial and virus strains**		

*Escherichia coli* BL21	TaKaRa	Cat# 9126
*Agrobacterium tumefaciens* EHA105	Yamada, T. et al.^73^	N/A

**Biological samples**		

Human nail fragment	Musashino university	N/A

**Chemicals, peptides, and recombinant proteins**		

NSC23766	Selleck Chemicals	Cat#S8031
MBQ-167	Selleck Chemicals	Cat#S8749
EHT1864	Cayman Chemical Company	Cat#17258
ML-141	Cayman Chemical Company	Cat#18496
EHop-016	Cayman Chemical Company	Cat#21557
ZCL-278	Cayman Chemical Company	Cat#14849
CASIN	Cayman Chemical Company	Cat#17694
Ketorolac	Cayman Chemical Company	Cat#9001148
Brefeldin A	Cayman Chemical Company	Cat#11861
NAV2729	TOCRIS Bioscience	Cat#5986
TrRac	This paper	N/A
TrCdc42-His	This paper	N/A
TrCdc24 DH-PH	This paper	N/A

**Critical commercial assays**		

NucleoSpin RNA	MACHEREY-NAGEL	Cat#740955
Quick-DNA Fungal/Bacterial Miniprep Kit	Zymo Research	Cat#D6005

**Deposited data**		

Trichophyton rubrum CBS 118892 rho guanyl nucleotide exchange factor mRNA, complete cds	This paper	GenBank: MW699015.1
HsCDC42 H. sapiens	NCBI Protein	NP_001034891.1
ScCDC42 S. cerevisiae S288C	NCBI Protein	NP_013330.1
CaCDC42 C albicans SC5314	NCBI Protein	XP_002417442.1
CnCDC42 C neoformans H99	NCBI Protein	XP_012053703.1
CnCDC420 C neoformans H99	NCBI Protein	XP_012050649.1
AnCDC42(ModA) A. nidulans FGSC A4	NCBI Protein	XP_026602456.1
AfModA A. fumigatus	NCBI Protein	XP_001260196.1
HsRac1 H. sapiens	NCBI Protein	NP_001003274.1
HsRac2 H. sapiens	NCBI Protein	NP_001248306.1
HsRac3 H. sapiens	NCBI Protein	NP_001092649.1
HsRhoG H. sapiens	NCBI Protein	AAM21121.1
AnRac1(RacA) A. nidulans FGSC A4	NCBI Protein	XP_662347.1
AfRac1 A. fumigatus	NCBI Protein	KAH1268728.1
CnRac1 C. neoformans H99	NCBI Protein	XP_012048200.1
CnRac2 C. neoformans H99	NCBI Protein	XP_012052960.1
CaRac1 C. albicans SC5314	NCBI Protein	XP_718826.2
ScRho5 S. cerevisiae	NCBI Protein	NP_014219.1

**Experimental models: Organisms/strains**		

*Trichophyton rubrum* CBS118892	White, T.C. et al.^74^	N/A
*Trichophyton rubrum* P_*ctr4*_*Trcdc24*	This paper	N/A
*Trichophyton rubrum* Δ*Trrac*	This paper	N/A
*Trichophyton rubrum* P_*ctr4*_*Trcdc42*	This paper	N/A
*Trichophyton rubrum* Δ*Trrac* P_*ctr4*_*Trcdc42*	This paper	N/A
*Bombyx mori*	Ehime-Sansh Co., Ltd.	Cat#Egg set

**Oligonucleotides**		

See Table S1 for primers.		

**Recombinant DNA**		

pGEX6p-1	Cytiva	28954648
pAg1P_*ctr4*_*Trcdc24*	This paper	N/A
pAg1Δ*Trrac*	This paper	N/A
pAg1P_*ctr4*_*Trcdc42*	This paper	N/A
pAg1	Zhang, A. et al.^75^	N/A
pAg 1−3′-UTR of ARB_02021	This paper	N/A
pAg1-*hph2*	Yamada, T. et al.^76^	N/A

**Software and algorithms**		

ImageJ	National Institutes of Health	ImageJ (RRID:SCR_003070)
MEGA X	Stecher, G. et al.^77^	X
Prism 10	GraphPad	10

### Resource availability

#### Lead contact

Further information and requests for resources and reagents should be directed to and will be fulfilled by the lead contact, Masaki Ishii (m_ishii@musashino-u.ac.jp).

#### Materials availability

All materials within the paper are available from the lead contact, Masaki Ishii (m_ishii@musashino-u.ac.jp), upon reasonable request.

#### Data and code availability

• The *Trcdc24* mRNA sequence has been registered in GenBank as MW699015.
• Further information and requests for resources and reagents should be directed to and will be fulfilled by the lead contact, Masaki Ishii (m_ishii@musashino-u.ac.jp).

### Experimental model and study participant details

#### Bacterial and fungal strains and culture conditions

*Trichophyton rubrum* CBS118892, *Escherichia coli* BL21, and *Agrobacterium tumefaciens* EHA105 were used.^74^^,^^78^^,^^79^ *E. coli* BL21 was cultured in LB medium with appropriate antibiotics at 37°C. *A*. *tumefaciens* was cultured in LB or *Agrobacterium* induction medium supplemented with 0.2 mM acetosyringone at 28°C.^73^ *T. rubrum* was cultured at 28°C. Conidia of *T. rubrum* were prepared using a previously described method.^80^ Sabouraud agar or RPMI with MOPS agar was used for hyphal formation. To evaluate mycelial growth inhibition by inhibitors, a modified 1/10 Sabouraud glucose agar (containing 0.2% bacto peptone, 0.1% glucose, 0.1% KH_2_PO_4_, 0.1% MgSO_4_ 7H_2_O, 1.5% bacto agar, pH unadjusted) was used.

### Method details

#### Inhibitors

NSC23766 and MBQ-167 were procured from Selleck Chemicals, Houston, TX, USA. EHT1864, ML-141, EHop-016, ZCL-278, CASIN, Ketorolac, and Brefeldin A were obtained from Cayman Chemical Company, Germany. AZA1, Rhosin, ITX3, and Y16 were sourced from Merck, Germany. NAV2729 was acquired from TOCRIS Bioscience, UK. All inhibitors were solubilized in DMSO.

#### T. rubrum conidia germination assay

Fungal conidia (2 × 10^6^) were subjected to an overnight incubation at 28°C in Sabouraud medium, supplemented with 1% DMSO (control) or the Rac and/or Cdc42 inhibitors; 10, 32, and 100 μM EHop-016; 100, 320, and 1000 μM AZA1; 10, 32, and 100 μM EHT1864; 10, 100, and 1000 μM CASIN; 200, 500, and 1000 μM NSC23766; 20, 200, and 2000 μM ketorolac; 100 μM MBQ-167; 1 and 10 μM ML-141; 1000 μM ZCL278; and 50 μM ITX3. Following the incubation, the fungal conidia were washed twice with saline and then stained with 0.2 mM calcofluor white. The germinated and ungerminated conidia were subsequently observed and counted under a fluorescent microscope (Olympus BX53). For the purpose of this experiment, conidia were considered germinated if they exhibited a protrusion that exceeded the diameter of the conidia.

#### Plasmid construction

The primers used in this study are listed in Table S1. *Trrac*, *Trcdc42*, and *Trcdc24*DH-PH were cloned into pGEX6p-1 using the In-Fusion system (TaKaRa Bio, Japan). The amplified DNAs were inserted into the EcoRI–BamHI sites of pGEX6p-1. To prepare TrCdc42-His, TrCdc42 containing pGEX6p-1 was used as the template. These sequences were sequenced post cloning.

A *Trcdc24*-targeting vector, pAg1P_*ctr4*_*Trcdc24*, was constructed as follows: approximately 1.8 and 1.9 kb of the upstream and ORF fragments of *Trcdc24* (TERG_02186) were amplified from *T. rubrum* total DNA through PCR with specific primer pairs. The basic structures of pAg1^75^ and Pctr4 cassette (P*trpC* [GenBank accession no. X02390], *nptII*, T*cgrA* [AFUA_8G02750], and P*ctr4* [TERG_01401]) were amplified from pAg 1−3′-UTR of ARB_02021 through PCR with specific primer pairs.

These four amplified fragments were joined with the In-Fusion system (TaKaRa Bio, Japan). Similarly, a *Trrac*-targeting vector, pAg1Δ*Trrac*, was constructed: approximately 1.6 and 1.0 kb of the upstream and downstream fragments of *Trrac* (TERG_02424) were amplified from *T. rubrum* total DNA through PCR with specific primer pairs. The basic structures of pAg and *Hygromycin B phosphotransferase* (*hph*) cassette (P*trpC* [GenBank accession no. X02390], *hph*, and T*cgrA* [AFUA_8G02750]) were amplified from pAg 1−3′-UTR of ARB_02021 and pAg1-*hph2*^76^ through PCR with specific primer pairs.

These four amplified fragments were joined with the In-Fusion system (TaKaRa Bio, Japan).

A *Trcdc42*-targeting vector, pAg1P_*ctr4*_*Trcdc42*, was constructed as follows: approximately 1.7 and 2.0 kb of the upstream and ORF + downstream fragments of *Trcdc42* (TERG_04946) were amplified from *T. rubrum* total DNA through PCR with specific primer pairs. The basic structures of pAg1^75^ and Pctr4 cassette (P*trpC* [GenBank accession no. X02390], *nptII*, T*cgrA* [AFUA_8G02750], and P*ctr4* [TERG_01401]) were amplified from pAg 1−3′-UTR of ARB_02021 through PCR with specific primer pairs. The ORF + downstream fragment and the linearized vector fragment containing upstream fragment were joined with the In-Fusion system (TaKaRa Bio, Japan). PCR was performed using Tks Gflex DNA polymerase (TaKaRa Bio, Japan).

#### Recombinant protein preparation

TrRac, TrCdc42-His, and TrCdc24 DH-PH proteins were expressed using *E. coli* BL21 carrying pGEX6p-1 derivative plasmids. *E. coli* BL21 carrying the plasmid was precultured in 5 mL of LB with 50 μg/mL ampicillin at 37°C. The preculture was then inoculated into 2 L of LB with 50 μg/mL ampicillin and cultured at 37°C for 3 h. Expression was induced with 0.1 mM IPTG, and the culture was maintained overnight at 20°C. During GST-TrCdc42-His induction, 4% ethanol was added. The purification of GST fusion proteins followed the instructions of GSTrap HP (GE Healthcare Japan, Japan). The purification buffer (Buffer A) consisted of 20 mM Tris/HCl (pH 7.5), 150 mM NaCl, 2.5 mM MgCl2, and 0.5 mM DTT. Purified fusion proteins were treated with PreScission protease at 4°C, and the flow-through of GSTrap was collected. After PreScission protease treatment, TrRac protein was desalted using Amicon UltraCel 10K (Merck, Germany), and the flow-through of Q Sepharose HP (GE Healthcare Japan, Japan) was recovered. Following pre-scission protease treatment, Cdc42-His protein was further purified using Ni-NTA resin (FUJIFILM Wako Chemicals, Japan) and desalted using Amicon UltraCel10K (Merck, Germany). For CDC42, without a His tag at the C-terminus, partial fragments of the protein were fractionated together with the full-length protein in the final fraction. Therefore, by fusing a His tag to the C-terminus of the protein and purifying it with a glutathione column in addition to an Ni-NTA column, we were able to achieve sufficient purity. The purity of the TrRac, TrCdc42-His, and TrCdc24 DH-PH proteins was analyzed using fluorescence staining and stain-free imaging technology from Bio-Rad Laboratories (Hercules, CA, USA).

#### Guanine nucleotide exchange assay

One μM of TrRac or TrCdc42-His was mixed with 0.1 μM bodipy-FL GTP (Thermo Fisher, Waltham, MA, USA) in Buffer A and allowed to equilibrate for 3 min at 20°C–25°C. Ammonium sulfate, EDTA, or recombinant TrCdc24 DH-PH proteins were incubated with the compounds or vehicles alone (final DMSO concentration: 1%) at the indicated concentrations in the reaction buffer for 20 min at room temperature. The reaction was initiated by combining TrRac or TrCdc42/bodipy-FL GTP (100 μL) with Cdc24DH-PH (50 μL) at room temperature. The change in bodipy-FL-GTP fluorescence (excitation: 485 nm, emission: 535 nm) was monitored for 60 min using a Berthold tristar2 LB942.

#### Transformation of T. rubrum

*T*. *rubrum* was transformed using the *A. tumefaciens*-mediated transformation (ATMT) method as previously described with minor modifications.^9^^,^^73^^,^^81^ After cocultivation, nylon membranes were transferred onto Sabouraud dextrose agar (SDA) containing 200 μg/mL G418 (Nacalai Tesque, Japan), chloramphenicol, and 200 μg/mL cefotaxime sodium. They were then overlaid with 10 mL of SDA supplemented with 400 μg/mL of G418, chloramphenicol, and 200 μg/mL cefotaxime sodium and incubated for 7–14 days. Transformants were subsequently transferred onto new SDA plates with appropriate antibiotics. The desired transformants were screened through PCR and Southern blot analysis. Total DNA was extracted using the Quick-DNA Fungal/Bacterial Miniprep Kit (Zymo Research, Irvine, CA, USA). Beads beating of fungal cells was performed using μT-01 (TAITEC, Japan) with 5 mm stainless steel beads. Aliquots of approximately 0.5–1 μg of the total DNA were digested with an appropriate restriction enzyme, separated via electrophoresis on 0.8% (w/v) agarose gels, and transferred to Hybond-N+ membranes (GE Healthcare Ltd., Chicago, IL, USA). Southern hybridization was performed using the ECL Direct Nucleic Acid Labeling and Detection System (GE Healthcare Ltd. , Chicago, IL, USA) following the manufacturer’s instructions.

#### Quantitative PCR

*Trichophyton rubrum* was precultured for 1 week in MOPS-buffered RPMI. The preculture was inoculated in MOPS-buffered RPMI with the desired concentration of CuSO_4_ or BCS. Harvested mycelium was homogenized using a bead shocker (TITEC, Japan). Total RNA was purified using NucleoSpin RNA (MACHEREY-NAGEL, Germany). Reverse transcription and PCR reactions were conducted as previously described.^82^^,^^83^ Primers used for the quantification of mRNA level are listed in Table S1.^84^

#### Cell morphological analysis

The cells were observed using a fluorescence microscope, specifically BX53 Japan. Subsequently, the measurements of cell length and width were conducted using ImageJ software, NIH. The polarity index was calculated as the length, which was measured at the longest diameter of a cell, divided by the width, measured at the midpoint of the length and perpendicular to the longest diameter, following the methodology outlined in a previous study.^75^

#### Indirect fluorescence microscopy

The P_*ctr4*_*Trcdc24* strain was inoculated with 1–5 × 10^6^ spores on sterile coverslips positioned in a 12-well plate. Subsequently, the fungal spores were incubated with 500 μL of SD liquid medium overnight at 28°C. On the second day, the SD medium was refreshed with a fresh medium, and the spores were incubated once again overnight at 28°C. On the third day, the supernatant was carefully removed, and the cells were fixed with 4% paraformaldehyde (PFA, sourced from Nacalai Tesque, Japan) for 15 min at room temperature. Following this, the samples were washed three times with PBST (PBS +0.05% Tween 20) and then incubated in 400 μL of 10 mg/mL lysing enzyme/10% bovine serum albumin (BSA)/PBS for 2 h at 28°C. Afterward, they were washed three times with PBST, permeabilized with 400 μL of precooled methanol at −25°C for 10 min, and then washed again with PBST. The samples were incubated with blocking buffer (10% normal donkey serum/0.2% Triton X-100/0.02% sodium azide/PBS) for 30 min, followed by incubation with anti-beta-actin antibody (clone C-4, Santa Cruz Biotechnology; diluted 1/1000) in Can Get Signal A solution (Toyobo, Japan) at 4°C overnight. Following three washes with PBST, the samples were incubated with Donkey Anti-Mouse IgG H&L (Alexa Fluor 488) preadsorbed (Abcam; diluted 1/1000) and DAPI solution (Dojindo, Japan; diluted 1/100000) in Can Get Signal A solution for 1 h at room temperature. After three additional washes with PBST, the samples were rinsed with water, mounted on glass slides using Aqua-Poly/Mount (Polysciences, UK), and finally observed under a BZ-8100 all-in-one fluorescence microscope (Keyence, Japan) or an AX confocal microscope system (Nikon, Japan).

#### Phylogenetic tree analysis

The evolutionary history of Cdc42 and Rac GTPases was deduced using the neighbor-joining method.^85^ The percentage of replicate trees in which the associated taxa clustered together in the bootstrap test (500 replicates) is depicted adjacent to the branches. Evolutionary distances were computed using the Poisson correction method and are presented as the number of amino acid substitutions per site. Positions containing gaps and missing data were excluded using the complete deletion option. Evolutionary analyses were conducted using MEGA X.^77^

#### Invertebrate infection model of dermatophytosis

Eggs from silkworms were procured from Ehime-Sansh Co., Ltd. (Japan) and reared following a method described previously.^86^^,^^87^ Fifth instar silkworm larvae were nourished with an artificial diet (Silkmate 2S; Ehime-Sanshu Co., Ltd., Japan) overnight.^88^^,^^89^ Dermatophyte infection was conducted in accordance with a previously outlined procedure.^53^ Conidia (5 × 10^6^) of *T. rubrum* were injected into the silkworm hemolymph.

#### *In vitro* nail infection assay

The conidial suspension (1 × 10^4^/10 μL/nail fragment) of *T. rubrum* was administered to autoclaved nail fragments obtained from an adult male volunteer (ethics approval, No. R3-6, Musashino University, Japan) and incubated at 28°C. After 24 h, either the vehicle (30% DMSO/5% Tween 80) or 60 mM EHop-016 was applied to the nail fragment. A total of four applications were administered every 24 h. The nails were examined after 36 days of incubation.

### Quantification and statistical analysis

#### Statistical analysis

Statistical analyses were conducted using Microsoft Excel and Prism 10, GraphPad. All experiments were performed more than three times. Statistical significance was determined using Student’s t test, one-way analysis of variance, and Tukey–Kramer test. IC_50_ values were ascertained by plotting the fluorescent intensity (% of control) at 60 min using a specific formula:

$$\text{I}C_{50}=x2\;-\;{\left(x2\;/\;x1\right)}^{\left(50\;-\;y2\;/\;y2\;-\;y1\right)}$$

x1: sample concentration lesser than IC_50_,

x2: sample concentration higher than IC_50_

y1: fluorescence intensity (%) at x1,

y2: fluorescence intensity (%) at x2.
