# Supplementary material for: Targeting dermatophyte Cdc42 and Rac GTPase signaling to hinder hyphal elongation and virulence
Source: iScience. 2024 May 28;27(6):110139. doi: 10.1016/j.isci.2024.110139 (PMC11215307; doi:10.1016/j.isci.2024.110139)
Supplement: Document S1. Figure S1 and Table S1 [file mmc1.pdf]

iScience, Volume *27*

Supplemental information

Targeting dermatophyte Cdc42 and Rac GTPase signaling to hinder hyphal elongation and virulence

Masaki Ishii, Yasuhiko Matsumoto, Tsuyoshi Yamada, Hideko Uga, Toshiaki Katada, and Shinya Ohata

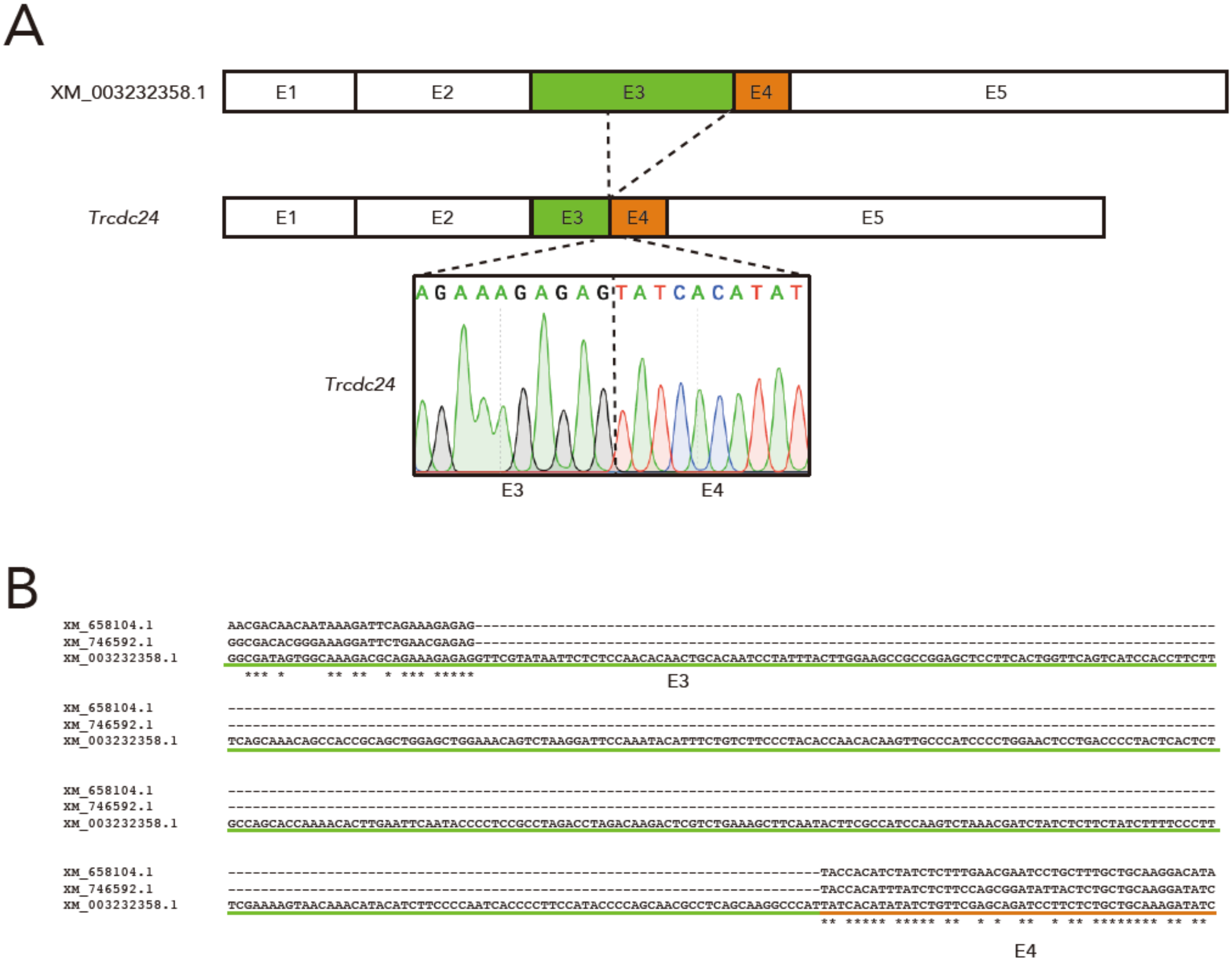

Figure S1

Figure S1 Part of XP_003232406.1 is a TrCdc42/TrRac GEF TrCdc24, related to Figure 3.

A. Exons of XM_003232358.1 coding XP_003232406 and *Trcdc24*.

B. Partial sequences of *cdc24* gene in A. nidulans (XM_658104.1), A. fumigatus (XM_746592.1) and XM_003232358.1 exon 3 and exon 4 in *T. rubrum*.

Table S1. Primers used in this study, related to the STAR Methods.

Primer	Sequence
**For vector construction**	
Trrac-F-pGEX	5'- GGG GCC CCT GGG ATC CAT GGC TTC TGG TCC AGC TAC -3'
Trrac-R-pGEX	5'- GTC GAC CCG GGA ATT CTA CTT TCT CTT TGG TTT CTG AGT TGG A -3'
Trcdc42-F-pGEX	5'- GGG GCC CCT GGG ATC CAT GGT TGT TGC TAC TAT CAA GTG TG -3'
Trcdc42-R-pGEX	5'- GGA ATT CCG GGG ATC CTA CAA GAG TAG ACA GCG GCT C -3'
Trcdc24-F-pGEX	5'- GGG GCC CCT GGG ATC ACT CGA GCT TCA AGG CCA AC -3'
Trcdc24-R-pGEX	5'- GTC GAC CCG GGA ATT CCG CTG CTT GTC GAT ATC CT -3'
pGEX6p-1-6HisTGA-F	5'- CAT CAC CAT CAC CAT CAC TGA CGA TCT GCC TCG C -3'
CDC42-6His-R	5'- ATG GTG ATG GTG ATG CAA GAG TAG ACA GCG GCT CG -3'
Trcdc24-5'-F	5'- GGG AAA CGA CAA TCT ATA ACC CCG GTG ATG GAG GA -3'
Trcdc24-5'-R	5'- TCA ATA TCA TCT TCT ACC CCC AAT AGC CAA ACT GG -3'
Trcdc24-orf-F	5'-TAC AAA GCC TGC GAA ATG GAG GGA ATG AAC GGG AA -3'
Trcdc24-orf-R	5'- GTG AAT TCG AGC TCG TGG AAA CTT GCT GGT CTG GT -3'
pAg1-F	5'- CGA GCT CGA ATT CAC TGG CC -3'
pAg1-R	5'- AGA TTG TCG TTT CCC GCC TT -3'
Pctr4-F	5'- AGA AGA TGA TAT TGA AGG AGC A -3'
Pctr4-R	5'-TTC GCA GGC TTT GTA CTT T -3'
Trrac-5'-F	5'- GGG AAA CGA CAA TCT ACA GCT GAG AAG GTC AAG GC -3'
Trrac-5'-R	5'- TCA ATA TCA TCT TCT TTT CCA GCG AAA ACA CCA GC -3'
Trrac-3'-F	5'- TAC AAA GCC TGC GAA CCA GCG ATC ATC GAC CTT GA -3'
Trrac-3'-R	5'- GTG AAT TCG AGC TCG AAG ATG GAG GTG GAT GGG GA -3'
Trcdc42-5'-F	5'- CGC ACT AGT GGG ATT TGG AGT CAA GGC GA -3'
Trcdc42-5'-R	5'- CGC GGG CCC CTC TCT CCT GCT GGT CTC CT -3'
Trcdc42-orf-F	5'- TAC AAA GCC TGC GAA CAT GGT TGT TGC TAC TAT CA -3'
Trcdc42-orf-R	5'- GTG AAT TCG AGC TCG GAA CGG GAC AGG TTC GAC TT -3'
pAg1-Trcdc42-orf-F	5'- CGA GCT CGA ATT CAC TGG CC -3'
pAg1-Trcdc42-orf-R	5'- TTC GCA GGC TTT GTA CTT T -3'
**For confirming genome structure**	
Primer 1	5'- ATG GCT TCT GGT CCA GCT AC -3'
Primer 2	5'- GCG CGT GAT GTT CGA TCT CG -3'
Primer 3	5'- AGA AGA TGA TAT TGA AGG AGC ACT TTT TGG GCT T -3'
Primer 4	5'- AGA TGA TTC ATG ACG TAT ATT CAC CG -3'
Primer 5	5'- GGG ATT TGG AGT CAA GGC GA -3'
**For RT-PCR**	
Trcdc24-RT-F	5'- ACA GAA CCG GTA CAC TGC CC -3'
Trcdc24-RT-R	5'- AAC GGA GGT AAT GAG GGC CG -3'
Trcdc42-RT-F	5'- TGG AGA TGA GCC ATA CAC GC -3'
Trcdc42-RT-R (Primer 6)	5'- TCT CAA AGG AAG CTG GCG AG -3'
rpb2-RT-F	5'- TGC AGG AGC TGG TGG AAG A -3'
rpb2-RT-R	5'- GCT GGG AGG TAC TGT TTG ATC AA -3'
